# SG-APSIC1038: Hospital-wide study to evaluate the tolerability and acceptability of alcohol-based hand rubs according to WHO protocol, and healthcare worker hand hygiene behavior during the COVID-19 pandemic

**DOI:** 10.1017/ash.2023.49

**Published:** 2023-03-16

**Authors:** Yew Fong Lee, Wei Hong Lai, Peh Yee Lee, Samual Chuo Yew Ting, Irena Albert Nuja, Hie Ung Ngian, Jiancong Wang

**Affiliations:** Sarawak General Hospital, Kuching, Malaysia; Clinical Research Centre, Institute for Clinical Research, National Institutes of Health, Kuching, Malaysia; Infection Prevention and Control Unit, Sarawak General Hospital Kuching, Malaysia; Pharmaceutical Services Division, Sarawak State Health Department, Kuching, Malaysia; Infection Prevention and Control Unit, Sarawak General Hospital, Kuching, Malaysia; Hospital Director’s Office, Sarawak General Hospital, Kuching, Malaysia; Clinic for Infectious Diseases and Hospital Hygiene, University Hospital Zurich, Zurich, Switzerland

## Abstract

**Objectives:** To evaluate the tolerability and acceptability of 3 different alcohol-based hand rub (ABHR) products, and to determine factors influencing hand hygiene (HH) behavior among healthcare workers (HCWs) during the COVID-19 pandemic. **Methods:** A cross-sectional study was conducted in Sarawak General Hospital, a 1,034-bed tertiary-care state hospital. A self-administered 7-point Likert scale questionnaire was adapted from the WHO ‘Protocol for Evaluation of Tolerability and Acceptability of ABHR.’ The study was conducted between November 12 and 26, 2021, based on 3 types of ABHR products. Participation in answering the questionnaire was voluntary, so consent was implied. The Student *t* test was used to determine the significant differences among the ABHR product. The χ^2^ distribution test was performed to evaluate the characteristics of ABHR products. **Results:** We received a response rate of 35% (1,598 of 4,628); 82% of respondents were female, and the overall cohort had a mean age of 35 years. Also, 972 (61%) of 1,598 respondents were nurses, and 1,490 (93%) of 1,598 respondents used ABHR at least 5 days every week. Of 1,598 respondents, 1,156 (72%) indicated that ABHR products were easily accessible at the point of patient care. Evaluation of ABHR products showed that respondents were receptive to all product colors (*P* < .0114) and had no color preference (*P* > .05). Comparison among ABHR products yielded no statistical difference (*P* > .05) for ‘smell,’ ‘stickiness,’ ‘irritation,’ or ‘drying speed.’ ‘Drying effect’ of all products was statistically significant (*P* < .0252). The overall satisfaction for all products was good (*P* < .0022). HCWs did not expect their HH compliance to improve even if they were provided with their preferred choice of ABHR. Of 1,598 respondents, 783 (49%) correctly used a palm-full of ABHR for HH, and 1, 275 (80%) indicated that hospital management should organize more HH-related awareness and continuous medical education on HH. **Conclusions:** A comparison among different ABHR characteristics mostly showed no statistically significant difference regarding tolerability and acceptability. These findings suggest that different ABHR products will not influence HH behavior during COVID-19 pandemic.

